# Polysialic acid is associated with better prognosis and IDH1-mutation in diffusely infiltrating astrocytomas

**DOI:** 10.1186/1471-2407-14-623

**Published:** 2014-08-28

**Authors:** Katri Mäkelä, Kristiina Nordfors, Jukka Finne, Anne Jokilammi, Timo Paavonen, Hannu Haapasalo, Miikka Korja, Joonas Haapasalo

**Affiliations:** School of Medicine, University of Tampere, Biokatu 6, 33520 Tampere, Finland; Department of Pathology, Fimlab Laboratories, Biokatu 4, PL 2000, 33521 Tampere, Finland; Department of Pediatrics, Tampere University Hospital, Teiskontie 35, 33521 Tampere, Finland; Department of Biosciences, Division of Biochemistry and Biotechnology, University of Helsinki, P.O.B. 56, FI 00014 Helsinki, Finland; Department of Medical Biochemistry and Genetics, University of Turku, Kiinamyllynkatu 10, 20520 Turku, Finland; Department of Neurosurgery, Helsinki University Central Hospital, P.O. Box 266, FI-00029 Helsinki, Finland; Australian School of Advanced Medicine, Macquarie University, 2 Technology Place, Suite 201, Sydney, NSW 2109 Australia; Unit of Neurosurgery, Tampere University Hospital, Teiskontie 35, 33521 Tampere, Finland

**Keywords:** Glioma, Astocytoma, Polysialic acid, PolySia, Neural cell adhesion molecule, IDH1, Survival

## Abstract

**Background:**

The aim of the study was to assess the localization of Polysialic acid (polySia) and Neural cell adhesion molecule (NCAM) in grade I–IV astrocytomas by confocal microscopy, and also to clarify and compare their relationship to conventional clinicopathological features in these tumors.

**Methods:**

Study material was stained immunohistochemically for polySia, NCAM and *IDH1-*R132H point mutation. Confocal microscopy of polySia and NCAM staining was performed on tissue micro-array samples (TMA) of 242 diffusely infiltrating astrocytomas (grade II: 28; grade III: 33; grade IV: 181) and 82 pilocytic astrocytomas. The results were statistically correlated to clinicopathological factors and survival data.

**Results:**

PolySia was observed in 45 cases (19%) and NCAM positivity in 92 cases (38%). All 45 tumors with polySia positivity were also positive for NCAM whereas there were 47 tumors which contained positive staining for NCAM but not for polySia. The simultaneous expression was concomitant and colocalized suggesting polysialyated NCAM (polySia-NCAM). PolySia expression was significantly stronger in *IDH1* mutated tumors than in *IDH1* non-mutated (p = 0.001, chi-square test). There were no significant differences in polySia-NCAM between primary tumors or recurrences (p = n.s., chi-square test). PolySia positivity was associated with longer patient survival in relation to total tumor material (p = 0.020, log-rank test). Furthermore, when only glioblastomas were assessed, patients with positive polySia had significantly better prognosis (p = 0.006, log-rank test). In multivariate survival analysis, polySia was found to be an independent prognostic factor. PolySia was nearly absent in grade I pilocytic astrocytomas (1 immunopositive tumor of 82).

**Conclusions:**

Expression of polySia is common in adult grade II–IV astrocytomas, whereas it is nearly absent in pediatric grade I pilocytic astrocytomas. PolySia positivity is associated with longer survival rates in patients with a grade II–IV astrocytomas and also grade IV glioblastomas assessed separately. The results of this study suggest that *IDH1* mutation may be associated with polySia expression pathways in malignant gliomas.

## Background

Diffusely infiltrating astrocytomas are central nervous system (CNS) tumors originating from astrocytic glial cells, or their precursors. They represent an important tumor entity accounting for 60% of all primary brain tumors [1]. Diffusely infiltrating astrocytomas can be assorted into grades II – IV according to WHO criteria. The most malignant type, glioblastoma (GBM), is the most prevalent form in adult patients. The devastating nature of GBM is highlighted by the 5-year survival rate of 10% even with using the latest therapeutical methods [2]. Lower grade astrocytomas most often relapse and proceed towards the more malignant grade. Primary GBMs most commonly occur as a new onset disease, whereas secondary GBMs develop from lower grade astrocytomas. Grade I astrocytomas are called pilocytic astrocytomas. They are considered benign as they have a clear borderline and are generally slow growing.

Neural cell adhesion molecule (NCAM) mediates adhesion between adjacent neurons and glial cells [3, 4]. Polysialic acid (polySia) is a carbohydrate polymer, which is added post-translationally to the extracellular part of NCAM, hence locating on the cell surface [5]. PolySia is a strongly hydrated and negatively charged molecule. By binding to NCAM, it increases the hydrodynamic area of the cells extern proximity and impairs the capability of NCAM to maintain the cell adhesion structures between other cells and the ECM [3, 6]. In normal tissues, especially during embryonic development, polySia takes part in neuronal cell migration and axon pathfinding [7]. In this way, polySia can facilitate cell migration and plasticity as well as increase the cell’s invasion capability [8]. In adulthood, polySia expression has mainly been associated with various pathologies, such as, for example, poor patient outcome and increasing WHO grade in astrocytic tumors [9, 10]. In contrast, NCAM expression has been shown to correlate with lower malignancy grade and better patient outcome [11, 12].

As polysialylated NCAM (polySia-NCAM) is considered to be a neural stem cell marker [13], and diffusely infiltrating astrocytomas are often referred as stem cell cancers [1], we studied for the first time whether the characteristics and clinical course of astrocytomas with and without polysialylated NCAM differ from each other. In addition, given that the isocitrate dehydrogenase 1 *(IDH1)* mutation has been found to be an essential genetic aberration in grade II–III diffusely infiltrating astrocytomas and especially in secondary GBMs [14, 15], we wanted to study whether this stem cell marker, polySia-NCAM, can be of additional help in identifying the prognostic characteristics if grade II-IV astrocytomas and secondary GBMs with and without IDH1 mutations.

## Methods

### Study material

There were 242 diffusely infiltrating astrocytomas (grade II: 28; grade III: 33; grade IV: 181) and 82 grade I pilocytic astrocytomas. 187 were primary astrocytomas and 55 were recurrences. Of the grade IV astrocytomas, 10 were gliosarcomas, 1 was a gliant cell glioblastoma and 170 were glioblastomas. Astrocytoma specimens were initially fixed in 4% phosphate-buffered formaldehyde and then processed into paraffin blocks. On the basis of hematoxylin and eosin-stained slides, a neuropathologist (H.H.) evaluated the tumors according to WHO 2007 criteria [1]. One histologically representative tumor region was selected from each specimen. From the selected regions, 1000 μm tissue cores were mounted into tissue microarray blocks using a custom made instrument (Beecher Instruments, Silver Spring, MD, USA). Samples were obtained from surgically operated patients at the Tampere University Hospital, Tampere, Finland, during the years 1983 to 2001. Tumors were radically resected if possible and most patients with high grade astrocytomas also received radiotherapy. Patient survival was examined by a follow-up study. Follow-up time started after primary resection of the astrocytoma. Patients’s progress was followed up to the year 2012 or until they were deceased. The study protocol was approved by the ethical committee of Tampere University Hospital and the National Authority for Medicolegal Affairs of Finland (diary number7796/05.01.00.06/2011).

PolySia-binding fluorescent fusion protein (EndoNA2-GFP) at a concentration of 10 μg/ml was used for polySia detection. Mouse anti-human NCAM antibody (123C3) at a concentration of 4 μg/ml (Santa Cruz Biotechnology, Santa Cruz, CA) was used as a primary antibody. Immunohistochemical incubations were done overnight at 4°C. In immunofluorescence, Alexa Fluor 594 chicken anti-mouse secondary antibodies (Molecular Probes, Eugene, OR) were used, and slides were mounted with Immu-Mount (Shandon, USA). Confocal microscopy was performed as described earlier [16]. The analysis was done for the TMA of the 242 paraffin-embedded diffusely infiltrating astrocytoma samples. A Leica TCS SP MP confocal microscope equipped with a Spectra-Physics Tsunami Ti-sapphire laser and Leica confocal software was used in analysis. Sections were examined at two excitation wavelengths: 488 nm for polySia-binding fusion protein EndoNA2-GFP and 546 nm for fluorescent secondary antibodies.

R132H point mutation specific mouse monoclonal antibody (Dianova GmbH, Hamburg, Germany) was used to detect *IDH1*-R132H specific gene mutations. Fully automated immunostaining was performed by a Bondmax immunostainer (Leica Biosystems Newcastle Ltd, Newcastle upon Tyne, United Kingdom). Bond Dewax Solution (catalogue No. AR9222) was used for deparaffinisation. For epitope retrieval, RTU Epitope Retrieval Solution 1, pH 5.9-6.1 (catalogue No. AR9961) was used for 30 min at 100°C. The slides were incubated for 30 minutes at room temperature with the *IDH1*-R132H point mutation specific antibody (dilution 1:50). The staining kit used was Bond Refine Detection kit. The slides were rinsed between steps with Bond Wash Solution (catalogue No. AR9590).

EGFR amplification status was examined by chromogenic in situ hybridisation (CISH) [17]. The cell proliferation index Ki-67/MIB-1 and p53 immunohistochemistry were performed and analysed as previously described [18, 19].

### Statistical analysis

The statistical analysis was performed using IBM SPSS Statistics version 20.0. Significant associations were defined using the chi-square test and Mann–Whitney U-test and Kruskall-Wallis test. Kaplan-Meier curves and log-rank test were used in univariate survival analyses. Cox Regression analysis was used for multivariate survival analyses.

## Results

**Figure 1:**
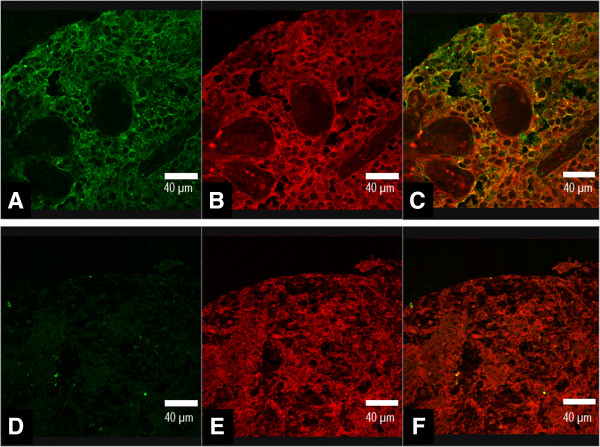
**Staining patterns of polysialic acid and NCAM.** A positive expression of polysialic acid detected with EndoNA2-GFP fusion protein is shown in figure **A**, and positive NCAM expression in figure **B** in glioblastoma. Overlay image of **A** and **B** showing the colocalized expression of polysialic acid and NCAM in same tumor sample **(C)**. Negative staining of polysialic acid **(D)**, positive staining for NCAM **(E)**, and overlay image of **D** and **E (F)** in pilocytic atrocytoma. Scale bar, 40 μm.

PolySia positivity was observed in 45 (18.6%) cases of 242 grades II – IV diffusely infiltrating astrocytomas. 92 (38.0%) tumors were shown to be positive for NCAM and 150 were negative. All tumors with polySia positivity were also shown to be positive for NCAM, whereas there were 47 tumors, which contained positive staining for NCAM but not for polySia. The simultaneous expression was concomitant and colocalized to cell surfaces indicating polysialylated NCAM. Figure 1 demonstrates the staining patterns of polySia and NCAM observed by confocal microscopy.

PolySia nor NCAM expression were not associated with WHO grade. Table 1 shows the numbers of polySia and NCAM positive tumors in each WHO grade. There was no correlation between polySia-NCAM expression and primary vs. recurrent tumors (p = n.s, chi-square test). PolySia was not associated with p53 expression or EGFR amplification (p = n.s., chi-square test).

**Table 1:** **Numbers of NCAM and polySia positive tumors in different WHO grades (p = n.s.)**

WHO grade				
	II	III	IV	Total
NCAM				
Positive	9 (32.2%)	18 (54.5%)	65 (35.9%)	92
Negative	19 (67.8%)	15 (45.5%)	116 (64.1%)	150
PolySia				
Positive	2 (7.1%)	8 (24.2%)	35 (19.3%)	45
Negative	26 (92.9%)	25 (75.8%)	146 (80.7%)	197

Interestingly, tumors having *IDH1* mutation were more often polySia-NCAM positive (p = 0.001, chi-square test): in *IDH1* mutated tumors there were 31 polySia positive and 18 negative cases, whereas, in *IDH1* non-mutated tumors, there were 120 positive and 19 negative cases. Similarly, NCAM positive tumors were more often *IDH1* mutated (p = 0.001, chi-square test; *IDH1* mutated: 20 negative and 29 positive for NCAM, *IDH1* non-mutated: 94 negative and 45 positive for NCAM). Positive polySia expression was also associated with increasing proliferation by MIB-1 / Ki-67 (p = 0.007, Kruskall-Wallis test).

**Figure 2:**
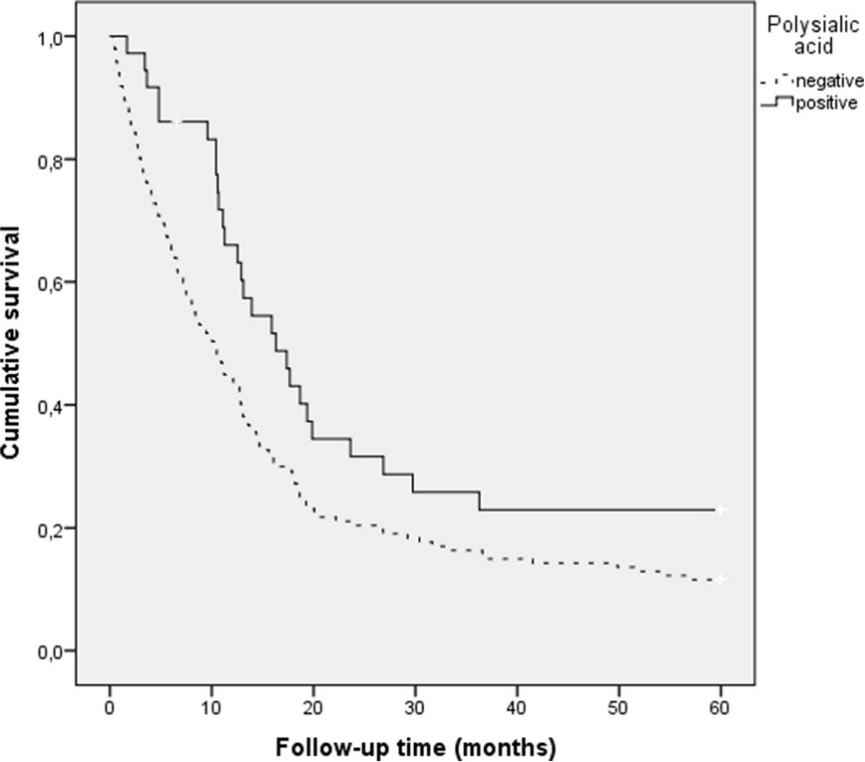
**Polysialic acid in grade II–IV astrocytomas (p = 0.020, log-rank test).** The patients with positive polySia had best prognosis. 183 patients were included in the analysis.

**Figure 3:**
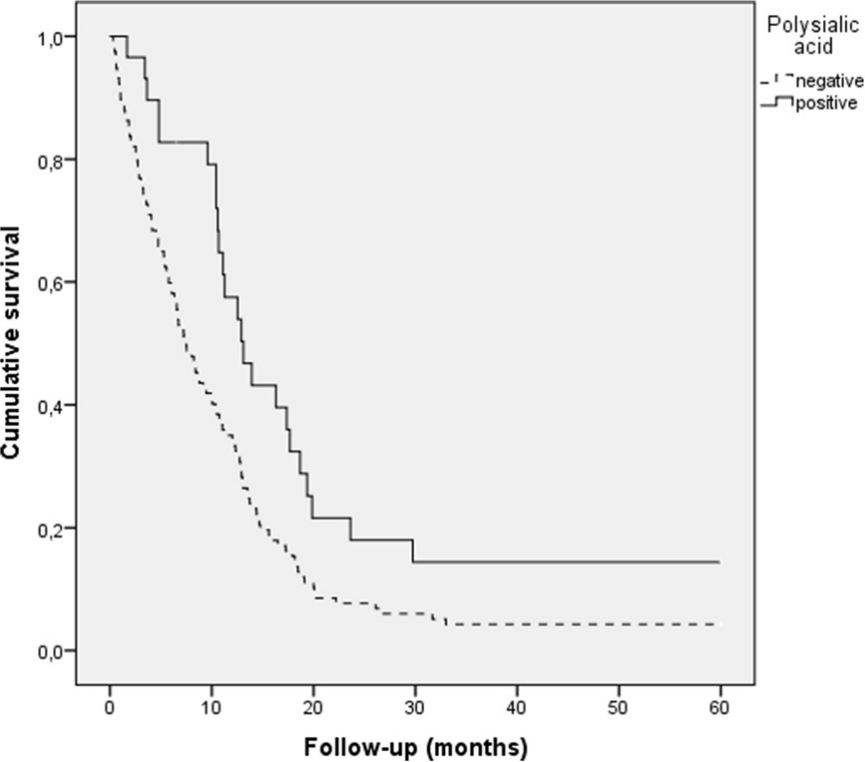
**Survival in glioblastomas by polysialic acid expression.** The patients with positive polySia-NCAM had significantly better prognosis (p = 0.006, log-rank test). 143 patients were included in the analysis.

Overall survival data was known for 187 patients. The median follow up time of survivors was 133 months. When all grade II – IV astrocytomas were included within the analysis, polySia positivity was associated with better patient prognosis (p = 0.020, log-rank test**,** Figure 2). In glioblastomas, positive polySia expression was associated significantly with better prognosis (p = 0.005, log-rank test, Figure 3). NCAM expression also associated with longer patient survival in total tumor material (p = 0.035, loq-rank test), but not within different grades, when studied separately. However, when only glioblastoma patients were analysed and both polySia and NCAM expression was assessed simultaneously, patients with both polySia and NCAM expression had longer survival rates than patients with negative polySia and positive or negative NCAM (p = 0.014, log-rank test). Furthermore, patients with NCAM expressing tumors had the worst prognosis compared to patients whose tumor was NCAM negative or expressed both NCAM and polySia. When grade II and III tumors were analysed separately, polySia or NCAM were not associated with patient prognosis (p = n.s, log-rank test).

Cox multivariate survival analysis was done with the following variables: patient age, IDH1 mutation, EGFR amplification, MIB1 labeling index, polySia expression, NCAM expression and WHO grade. There were 77 cases in which all these variables were known. The analysis was not done separately in each WHO grade because the cases included would have been few. IDH1 mutation, patient age, MIB1 and polySia expression came up as independent prognosticators. The hazard ratio (Exp(B)) for IDH1 was 0.195 (95% CI for Exp(B) 0.101-0.376). Exp(B) for patient age was 1.705 (95% CI for Exp(B) 1.217-2.390). Exp(b) for MIB1 was 1.673 (95% CI for Exp(B) 1.210-2.312). Exp(B) for polySia was 0.516 (95% CI for Exp(B) was 0.280-0.951). Age cut off poits were 0–54, 55–69, 70-. Cut off points for MIB1 were 0–5, 5.1-15, 15.1-.

### Polysialic acid in pilocytic astrocytomas

To assess the expression of polySia in grade I pilocytic astrocytomas, we conducted analysis on those mostly pediatric and benign tumors. Only one (1.2%) of the studied 82 tumors expressed polySia. In this case, polySia expression was located on the cell surfaces and it was histologically associated with NCAM (polySia-NCAM). NCAM positivity was detected in 22 tumors. Staining patterns of pilocytic astrocytoma is demonstrated in Figure 1.

## Discussion

PolySia is commonly expressed in grade II – IV astrocytomas and it is bound to NCAM, whereas it is nearly absent in grade I pilocytic astrocytomas. Patients with polySia expressing astrocytomas had better prognosis than patients with polySia negative astrocytomas. Furthermore, when only glioblastomas were studied, patients with tumors of polySia-NCAM positivity had similarly better prognosis. In multivariate survival analysis polySia came up as an independent prognostic factor, indicating better prognosis. Finally, polySia expression associated with one of the key features in gliomas, *IDH1* mutation. When *IDH1* mutation and polySia expression were assessed simultaneously, the patients with both polySia expressing and *IDH1* mutatated tumors had the best prognosis. Although polySia was associated with better prognosis in univariate and multivariate analysis, it was also associated with increasing proliferation. This underlines that mechanisms in protein expressions are complex.

Association of polySia expression with malignant tumors such as astrocytomas has been reported previously [9, 10]. However, the prognostic significance of histologically evaluated polySia expression in survival analyses was studied here for the first time. Similar to our findings in astrocytomas, polySia-NCAM expression is associated with better prognosis in neuroblastomas [16], which are also heterogenous tumors with stem cell-like characteristics. As speculated before, polySia-NCAM as a neural stem cell marker provides tumor cells not only with proliferative and mitotic characteristics but also with differentiation capability, like in the developing brain, thus improving prognosis [16]. Interestingly, patients with astrocytomas expressing only NCAM but not polySia had even worse prognosis than patients whose tumors were negative for both NCAM and polySia. Whether polySia expression prevents unfavorable effects of NCAM, such as leading cells towards epithelial-mesenchymal transition (EMT), or repress the intercellular connections mediated by NCAM through creating a hydrophilic radius on the cell surface, is speculative, but polySia expression may be an additional prognostic marker in most unfavorable astrocytomas, like in most unfavorable neuroblastomas [16].

Previously, Petridis et al. [9] as well as Amoureux et al. [10], have presented correlations between polySia expression and tumor malignancy in astrocytomas. In the study of Petridis et al., biopsies from 30 patients with astrocytomas I – IV were evaluated. The statistical analyses were performed combining grades I and II astrocytomas as one group and grades III and IV as another. They showed that higher grade astrocytomas were more often polySia positive. Given that we found only one out of 82 pilocytic astrocytomas as polySia-NCAM positive, the previous finding is not surprising. Since the biological background of pilocytic astrocytomas differs from malignant astrocytomas, the finding of Petridis et al. could be biased. However, for clinical purposes, it may be of use to use polySia-NCAM antibodies in immunohistochemical stainings of astrocytomas, since positive staining very likely rules out the possiblity of pilocytic astrocytomas. In this study, larger patient population, longer patient follow up time, confocal microscopy describing the anatomical location, and importantly, assessment of IDH mutation might provide additional value to the analysis.

Petridis et al. also suggested in their study that the pathways of regulation of polySia expression are mostly unknown and should be examined in future studies. In our study, polySia expression was shown to be associated with *IDH1* mutation, one of the key features of astrocytomas. Interestingly, *IDH1* mutation has recently been linked to EMT. In EMT, cells transform to a mesenchymal-like form and loose e-cadherin mediated cell-to-cell adhesions. EMT enables epithelial tumors to progress into metastatic cancers. Grassian et al. showed that high levels of the oncometabolite 2-HG, produced by mutant IDH1 enzyme, cause an epithelial-mesenchymal transition -like phenotype in *IDH1* mutated cells by changing the EMT-related gene expression and cellular morphology [20]. Also, both increased NCAM and polySia expression have been connected to EMT mainly via causing loss of the cell-to-cell adhesion molecule e-cadherin [21–23]. Thus, it is possible that *IDH1* mutation takes part in the regulation pathways of polySia-NCAM expression and should perhaps be further studied as a controlling factor of polySia-NCAM expression. Also, since polySia-NCAM has been proposed to be a marker of stem cells, another interesting theme for future research would be to study the expression of polySia and NCAM and their colocalisation in specific cellular subpopulations of diffusely infiltrating astrocytomas.

## Conclusion

PolySia expression in astrocytomas, and remarkably, in glioblastomas, was associated with longer overall survival rates. Since polySia expression is significantly more common in *IDH1* mutated tumors, and since these tumors have better overall prognosis, polySia may be a new additional molecular marker in the prognostic caharcterization of glioblastomas.

## Abbreviations

CNS: Central nervous system
GBM: Glioblastoma multiforme
IDH1: Isocitrate dehydrogenase 1
NCAM: Neural cell adhesion molecule
ECM: Extracellular matrix
polySia: Polysialic acid
EMT: Epithelial-mesenchymal transition
2-HG: 2-hydroxyglutarate.

## References

[1] Louis DN, Ohgaki H, Wiestler OD, Cavenee WK (2007). WHO Classification of Tumours of the Central Nervous System.

[2] Stupp R, Hegi ME, Mason WP, van den Bent MJ, Taphoorn MJ, Janzer RC, Ludwin SK, Allgeier A, Fisher B, Belanger K, Hau P, Brandes AA, Gijtenbeek J, Marosi C, Vecht CJ, Mokhtari K, Wesseling P, Villa S, Eisenhauer E, Gorlia T, Weller M, Lacombe D, Cairncross JG, Mirimanoff RO (2009). Effects of radiotherapy with concomitant and adjuvant temozolomide versus radiotherapy alone on survival inglioblastoma in a randomised phase III study: 5-year analysis of the EORTC-NCIC trial. Lancet Oncol.

[3] Seifert A, Glanz D, Glaubitz N, Horstkorte R, Bork K (2012). Polysialylation of the neural cell adhesion molecule: interfering with polysialylation and migration in neuroblastoma cells. Arch Biochem Biophys.

[4] Dallérac G, Rampon C, Doyère V (2013). NCAM function in the adult brain: lessons from mimetic peptides and therapeutic potential. Neurochem Res.

[5] Finne J, Finne U, Deagostini-Bazin H, Goridis C (1983). Occurrence of alpha 2–8 linked polysialosyl units in a neural cell adhesion molecule. Biochem Biophys Res Commun.

[6] Rutishauser U (1998). Polysialic acid at the cell surface: biophysics in service of cell interactions and tissue plasticity. J Cell Biochem.

[7] Brusés JL, Rutishauser U (2001). Roles, regulation, and mechanism of polysialic acid function during neural development. Biochimie.

[8] Suzuki M, Suzuki M, Nakayama J, Suzuki A, Angata K, Chen S, Sakai K, Hagihara K, Yamaguchi Y, Fukuda M (2005). Polysialic acid facilitates tumor invasion by glioma cells. Glycobiology.

[9] Petridis AK, Wedderkopp H, Hugo HH, Maximilian Mehdorn H (2009). Polysialic acid overexpression in malignant astrocytomas. Acta Neurochir (Wien).

[10] Amoureux MC, Coulibaly B, Chinot O, Loundou A, Metellus P, Rougon G, Figarella-Branger D (2010). Polysialic acid neural cell adhesion molecule (PSA-NCAM) is an adverse prognosis factor in glioblastoma, and regulates olig2 expression in glioma cell lines. BMC Cancer.

[11] Todaro L, Christiansen S, Varela M, Campodónico P, Pallotta MG, Lastiri J, Sacerdote de Lustig E, Bal de Kier Joffé E, Puricelli L (2007). Alteration of serum and tumoral neural cell adhesion molecule (NCAM) isoforms in patients with brain tumors. J Neurooncol.

[12] Duenisch P, Reichart R, Mueller U, Brodhun M, Bjerkvig R, Romeike B, Walter J, Herbold C, Regenbrecht CR, Kalff R, Kuhn SA (2011). Neural cell adhesion molecule isoform 140 declines with rise of WHO grade in human gliomas and serves as indicator for the invasion zone of multiform glioblastomas and brain metastases. J Cancer Res Clin Oncol.

[13] Pennartz S, Belvindrah R, Tomiuk S, Zimmer C, Hofmann K, Conradt M, Bosio A, Cremer H (2004). Purification of neuronal precursors from the adult mouse brain: comprehensive gene expression analysis provides new insights into the control of cell migration, differentiation, and homeostasis. Mol Cell Neurosci.

[14] Parsons DW, Jones S, Zhang X, Lin JC, Leary RJ, Angenendt P, Mankoo P, Carter H, Siu IM, Gallia GL, Olivi A, McLendon R, Rasheed BA, Keir S, Nikolskaya T, Nikolsky Y, Busam DA, Tekleab H, Diaz LA, Hartigan J, Smith DR, Strausberg RL, Marie SK, Shinjo SM, Yan H, Riggins GJ, Bigner DD, Karchin R, Papadopoulos N, Parmigiani G, Vogelstein B, Velculescu VE, Kinzler KW (2008). An integrated genomic analysis of human glioblastoma multiforme. Science.

[15] Balss J, Meyer J, Mueller W, Korshunov A, Hartmann C, von Deimling A (2008). Analysis of the IDH1 codon 132 mutation in brain tumors. Acta Neuropathol.

[16] Korja M, Jokilammi A, Salmi TT, Kalimo H, Pelliniemi TT, Isola J, Rantala I, Haapasalo H, Finne J (2009). Absence of polysialylated NCAM is an unfavorable prognostic phenotype for advanced stage neuroblastoma. BMC Cancer.

[17] Tanner M, Gancberg D, Di Leo A, Larsimont D, Rouas G, Piccart MJ, Isola J (2000). Chromogenic in situ hybridization: a practical alternative for fluorescence in situ hybridization to detect HER-2/neu oncogene amplification in archival breast cancer samples. Am J Pathol.

[18] Sallinen PK, Haapasalo HK, Visakorpi T, Helén PT, Rantala IS, Isola JJ, Helin HJ (1994). Prognostication of astrocytoma patient survival by Ki-67 (MIB-1), PCNA, and S-phase fraction using archival paraffin-embedded samples. J Pathol.

[19] Haapasalo H, Sallinen S, Sallinen P, Helén P, Jääskeläinen J, Salmi TT, Paetau A, Paljärvi L, Visakorpi T, Kalimo H (1999). Clinicopathological correlation of cell proliferation, apoptosis and p53 in cerebellar pilocytic astrocytomas. Neuropathol Appl Neurobiol.

[20] Grassian AR, Lin F, Barrett R, Liu Y, Jiang W, Korpal M, Astley H, Gitterman D, Henley T, Howes R, Levell J, Korn JM, Pagliarini R (2012). Isocitrate dehydrogenase (IDH) mutations promote a reversible ZEB1/microRNA (miR)-200-dependent epithelial-mesenchymal transition (EMT). J Biol Chem.

[21] Lehembre F, Yilmaz M, Wicki A, Schomber T, Strittmatter K, Ziegler D, Kren A, Went P, Derksen PW, Berns A, Jonkers J, Christofori G (2008). NCAM-induced focal adhesion assembly: a functional switch upon loss of E-cadherin. EMBO J.

[22] Frame MC, Inman GJ (2008). NCAM is at the heart of reciprocal regulation of E-cadherin- and integrin-mediated adhesions via signaling modulation. Dev Cell.

[23] Schreiber SC, Giehl K, Kastilan C, Hasel C, Mühlenhoff M, Adler G, Wedlich D, Menke A (2008). Polysialylated NCAM represses E-cadherin-mediated cell-cell adhesion in pancreatic tumor cells. Gastroenterology.

[CR24] The pre-publication history for this paper can be accessed here:http://www.biomedcentral.com/1471-2407/14/623/prepub

